# DGKα in Neutrophil Biology and Its Implications for Respiratory Diseases

**DOI:** 10.3390/ijms20225673

**Published:** 2019-11-13

**Authors:** Gianluca Baldanzi, Mario Malerba

**Affiliations:** 1 Center for Translational Research on Autoimmune & Allergic Diseases–CAAD, C.so Trieste 15/A, 28100 Novara, Italy; 2 Department of Translational Medicine, University of Eastern Piedmont 13100, Vercelli, Italy; 3 Respiratory Unit, Sant’Andrea Hospital, 13100 Vercelli, Italy

**Keywords:** lipid kinase, cell activation, tissue damage, signaling pathways

## Abstract

Diacylglycerol kinases (DGKs) play a key role in phosphoinositide signaling by removing diacylglycerol and generating phosphatidic acid. Besides the well-documented role of DGKα and DGKζ as negative regulators of lymphocyte responses, a robust body of literature points to those enzymes, and specifically DGKα, as crucial regulators of leukocyte function. Upon neutrophil stimulation, DGKα activation is necessary for migration and a productive response. The role of DGKα in neutrophils is evidenced by its aberrant behavior in juvenile periodontitis patients, which express an inactive DGKα transcript. Together with in vitro experiments, this suggests that DGKs may represent potential therapeutic targets for disorders where inflammation, and neutrophils in particular, plays a major role. In this paper we focus on obstructive respiratory diseases, including asthma and chronic obstructive pulmonary disease (COPD), but also rare genetic diseases such as alpha-1-antitrypsin deficiency. Indeed, the biological role of DGKα is understudied outside the T lymphocyte field. The recent wave of research aiming to develop novel and specific inhibitors as well as KO mice will allow a better understanding of DGK’s role in neutrophilic inflammation. Better knowledge and pharmacologic tools may also allow DGK to move from the laboratory bench to clinical trials.

## 1. Introduction

In this review we summarize the rapidly increasing body of knowledge that links diacylglycerol kinases (DGKs) to chronic respiratory diseases. DGKs are lipid kinases that modulate receptor signaling but also contribute to membrane trafficking and shaping. As neutrophils play a key role in chronic respiratory diseases, this article focuses on the numerous, but underappreciated, studies that indicate DGKs, and specifically the α isoform, as key regulators of the neutrophil life cycle.

## 2. The Diacylglycerol Kinase Family

DGKs are intracellular lipid kinases that phosphorylate diacylglycerol (DAG) to phosphatidic acid (PA). In mammals, ten DGK coding genes have been identified and classified into five different subtypes based on the presence of specific regulatory domains [1]. The presence of multiple genes and several alternative splicing events increases DGK family diversity, leading to a multiplicity of isoforms with distinct domain structures and expression patterns [2].

In the C-terminal portion, all isoforms feature a bipartite catalytic domain that identifies this family of enzymes. Unfortunately, this catalytic domain has never been structurally determined. However, it contains an ATP binding site where the mutation of a glycine to an aspartate or alanine renders the DGK kinase dead [3,4]. In addition to the catalytic domain, all DGK isoforms also contain at least two cysteine-rich domains, a feature homologous to the C1 domain of protein kinase C (PKC), which binds to phorbol-ester and DAG [5]. These C1 domains were initially suggested to participate in substrate recognition, however, they are not absolutely required for catalytic activity [6]. The C1 domain proximal to the catalytic domain has an extended region of fifteen amino acids not present in the C1 domains of other proteins, nor in the other C1 domains of the DGKs. This extended C1 domain somehow contributes to DGK activity, because mutations or the deletion of this domain significantly reduce the kinase activity of the enzyme [3]. Surprisingly, only the C1 domains of β and γ DGKs bind the DAG phorbol-ester analogues [7,8], suggesting that the C1 domains of the other isoforms putatively act in protein–protein interactions or in regulatory functions [5].

**Figure 1:**
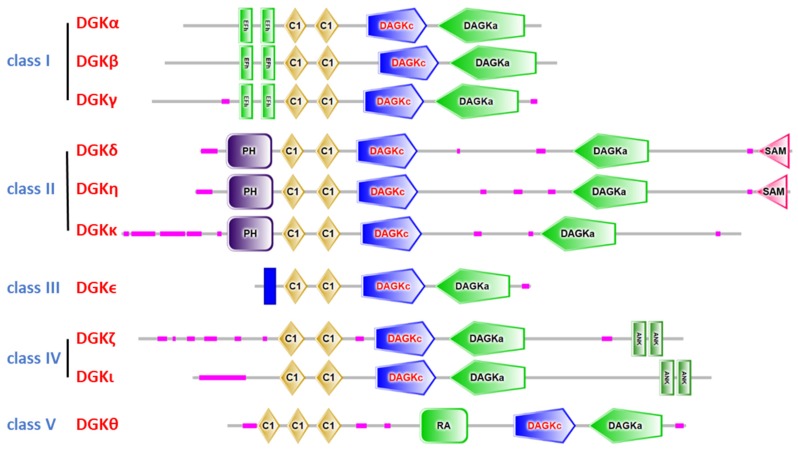
Structure of mammalian diacylglycerol kinases (DGKs). All DGKs share a conserved catalytic domain composed of a catalytic (DAGKc) and an accessory (DAGKa) subdomain, preceded by two or three C1 domains. Isoform-specific regulatory domains include EF hands, the pleckstrin homology domain (PH), Ras association domain (RA), sterile alpha motif (SAM), and ankyrin repeats (ANK). Low-complexity regions are shown in pink. Domain annotation by SMART [9].

Conversely, a significant divergence between the isoforms instead exists in the N-terminal regulatory domains, allowing to divide them into five classes on the basis of structural homology (Figure 1).

Class I—DGKα, DGKβ, and DGKγ are characterized by a conserved *N*-terminal recoverin homology domain and two calcium-binding EF hand motifs regulating membrane association and activity [10]. Recent structural studies have illustrated how calcium binding to the EF hand of DGKα removes an intramolecular interaction with the C1 domain, allowing the transition to an “open” active conformation [11,12].

Class II—DGKδ, DGKη, and DGKκ are characterized by an N-terminal plekstrin homology (PH) domain mediating the interaction with phosphatidylinositol 4,5-bisphosphate [13] and, putatively, proteins. In addition to the PH domain, DGKδ and DGKη also contain a sterile α motif (SAM) at their carboxy terminals capable of zinc-dependent oligomerization but also modulates their membrane localization [14]. Conversely, DGKκ lacks a SAM domain, but it does contain a C-terminal motif that may bind type I PDZ domains [15].

Class III—DGKϵ has an *N*-terminal hydrophobic α helix, preceding its tandem C1 domains, which is responsible for endoplasmic reticulum localization [16]. Interestingly, DGKϵ is peculiarly selective for poly-unsaturated fatty acids in position 2 of DAG and permanently associates to the membrane [17]. The constant activity of this isoform contributes to the enrichment of poly-unsaturated fatty acids in the phosphoinositide pool. Recessive mutations in DGKε results in hemolytic-uremic syndrome, probably due to reduced availability of poly-unsaturated fatty acids for prostanoid synthesis [18].

Class IV—DGKζ and DGKι contain a myristoyilated alanine-rich protein kinase C substrate domain (MARKS), which regulates protein–protein interactions and enzyme activity in a PKC-dependent manner [19,20]. Class IV DGKs also contain a PDZ-binding domain and ankyrin repeats responsible for protein–protein interactions [21].

Class V—DGKθ contains an N-terminal proline-rich domain, a third cysteine-rich domain, a PH domain, and a putative Ras-association domain within it [22].

The various mammalian DGKs show remarkably specific expression patterns, with most cells expressing multiple DGKs. Often, when several DGKs are expressed in tissues or cells, they are from different subfamilies, suggesting that each subfamily carries out a distinct biological function [23].

The majority of information on DGK’s biological function in neutrophils comes from studies with inhibitors or patients. Indeed, despite the availability of KO mice for DGKα [24], DGKβ [25], DGKδ [26], DGKε [27], DGKη [28], DGKι [29], and DGKζ [30], eventual alterations in neutrophil functions have not been investigated in animal models.

Actual knowledge is based on two commercially available DGK inhibitors (named R59949 and R59022), which are widely used in vitro [31,32]. They are reported to be selective for calcium-dependent type I DGK isozymes, and especially DGKα, with IC50 values of 25 and 18 μM, respectively, for R59022 and R59949 [33,34]. However, their use in vivo is severely limited by their reduced solubility and strong albumin binding [31,35,36]. Several groups attempted to overcome these limitations. Ritanserin was identified by us and others as a novel inhibitor of DGKα, with an IC50 around 20 μM based on its structural similarity to R59022 [37]. For this class of inhibitors, structural studies support a contiguous ligand-binding site composed of C1, DAGKc, and DAGKa domains in the DGKα active site [38,39]. However, the interpretations of data obtained with ritanserin, R59022, and R59949 are complicated by the observation that they all are combined pharmacological inhibitors of both DGKα and serotonin receptors in vitro [37]. Ritanserin was indeed originally characterized as a serotonin receptor antagonist and underwent clinical trials as a potential medicine for the treatment of schizophrenia and substance dependence [40,41,42,43,44,45,46,47,48,49,50,51,52]. Thus, ritanserin is highly promising for drug repurposing, as it is safe for human use at concentrations sufficient to inhibit DGKα, but it is poorly specific [53]. Interestingly, still starting from the two commercial inhibitors, we have recently characterized AMB639752, a novel DGKα-specific inhibitor deprived of serotonin antagonistic activity [54]. Finally, CU-3 is a promising compound that selectively inhibits DGKα, with an IC50 value of 0.6 µM by acting as an ATP competitive inhibitor without displacing DAG or phosphatidylserine [55]. However, the structure and reactivity of CU-3 makes its use unlikely in vivo. Thus, the pharmacological tools available mainly allow the study of DGKα functions, while studies on inhibitors that target the other DGK isoforms are still lacking. Moreover, the results obtained may be influenced by the poor selectivity of commercially available inhibitors.

## 3. DGKα in Neutrophil Biology

The DAG-mediated activation of conventional PKC and RASGRP4 are essential steps in neutrophil signal transduction, which leads to the activation of NADPH oxidase activity, cell movement, and extracellular trap release [56,57]. Conversely, the PA produced by DGKs (and also phospholipase D) promotes cell adhesion and degranulation [58,59]. Interestingly, in cell-free systems, the activation of neutrophil’s NADPH oxidase by DAG requires its conversion to PA by an R59022-sensitive DGK activity [60]. PA binds to the NADPH oxidase p47 subunit, together with phosphatidylinositol 3,4-bisphosphate, regulating membrane translocation after PKC-mediated phosphorylation [61].

Despite the relevance of the lipids metabolized by DGKs, their role in neutrophil differentiation is poorly known. In the HL-60 model cell line, only DGKα, DGKδ, DGKε, DGKγ, and DGKζ were expressed. Notably, DGKα was virtually absent in undifferentiated cells but was strongly upregulated throughout differentiation. Conversely, DGKε, DGKγ, and DGKζ were expressed in undifferentiated HL-60 cells but were strongly downregulated throughout differentiation. The inhibition of DGKα with R59022 and R59949 led to an acceleration of differentiation and concomitant cell cycle arrest [62]. This suggests that DGKs, and DGKα in particular, may play a relevant role during neutrophil differentiation. Conversely, in the same cell model, DGKγ negatively controlled differentiation toward the macrophage lineage, suggesting an isoform-specific biological role of DGKs during cell linage commitment [63].

**Figure 2:**
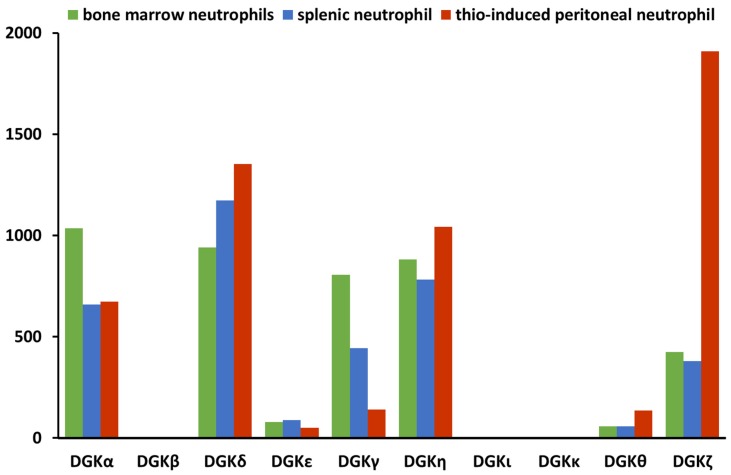
Variations in DGK family expressions in human neutrophils. Data from the immunological genome project, www.immgen.org.

Mature neutrophils express several DGK isoforms that undergo selective regulation during activation. Indeed, all isoforms, apart from DGKβ and DGKι, are detectable by RT-PCR in neutrophils [64]. Abundant expressions of DGKα, DGKδ, and DGKγ in human neutrophils have been reported by Oyaizu [65]. Microarray data (Immunological genome project, www.immgen.org) indicate that, in mice, DGKα and DGKγ are expressed by bone marrow granulocytes more than in splenic- or thiourea-induced peritoneal neutrophils. Here, DGKδ, DGKθ, and DGKζ are upregulated, while DGKε and DGKη are substantially stable (Figure 2 based on data from [66]).

In differentiated human neutrophils, the DGKα inhibitor R59022, at 10 μM, consistently enhances superoxide generation upon stimulation with fMet-Leu-Phe (fMLP), IgG, opsonized zymosan, LTB4, or IL-8 [67,68]. This activation is specific, as the release of lysozyme and *N*-acetyl-beta-glucosaminidase production triggered by fMLP is not stimulated by R59022. In vitro, the stimulation of human neutrophils with fMLP induces a large production of DAG, which is essential for downstream signaling. This DAG is metabolized largely by DGKα, and the diacylglycerol kinase inhibitor R59022 increases its concentration, indicating that, among DGKs, the α isoform plays a core role [69,70]. This central role of DGKα is due to selective activation in the membrane compartment by fMLP itself [71]. The retention of DAG as a result of DGKα inhibition induces a marked stimulation of superoxide production and potentiates arachidonic acid release via protein kinase C activation [72,73]. Paradoxically, by stimulating PKCs, the diacylglycerol kinase inhibitor R59022 also induces chemotaxis in resting neutrophils [74].

Multiple genetic defects affecting all stages of neutrophil development, trafficking, transmigration into tissues, and, in some instances, function, have been linked to exceptionally aggressive forms of familial periodontitis, which is a disorder characterized by an exaggerated inflammatory response in the oral mucosa, leading to the destruction of soft tissues and tooth-supporting bone in susceptible individuals [75].

Impaired neutrophil chemotaxis, exaggerated adhesion, and oxidative burst are observed in localized aggressive periodontitis (LAP, also known as localized juvenile periodontitis). Interestingly, DAG is elevated in unstimulated peripheral blood neutrophils from LAP patients. These cells also showed an enhanced and prolonged elevation of DAG in response to fMLP or zymosan, due to a decreased metabolism by DGK [76,77]. Significantly reduced chemotactic response, increased adhesion, and enhanced respiratory burst activity are also observed in R59022-treated normal neutrophils, suggesting an impairment in DGKα activity in LAP cells [78,79]. Indeed, the diminished RNA expression of DGKα in neutrophils from LAP patients causes this decreased DGK activity and the accumulation of DAG [80]. Further molecular studies identified a DGKα transcript that lacks exon 10 (DGKαΔ10). DGKαΔ10 features a premature stop codon and encodes a membrane-localized, truncated protein, which is upregulated in LAP neutrophils. The transfection of HL-60 neutrophil-like cells with the DGKαΔ10 splice variant induced an increase in the fMLP-stimulated production of superoxide anions, replicating the phenotype of LAP neutrophils [81]. These findings support the hypothesis that at least a subset of LAP is due to aberrant overexpression of the truncated DGKαΔ10 and impairment of DAG metabolism in neutrophils. The molecular links between reduced DGKα activity and reduced neutrophil-mediated immune defects are not clearly established but are generally attributed to excessive PKC activity. However, as we will discuss in this review, PA and DAG may regulate multiple components in the neutrophil signaling pathway, suggesting further research on this subject.

Anti-neutrophil cytoplasmic antibodies (ANCAs) are autoantibodies (predominantly immunoglobulin G) directed against constituents of neutrophil’s primary granules and lysosomes of monocytes. Among the several ANCA antigenic targets identified until now, the two more clinically relevant and best-characterized are myeloperoxidase and proteinase 3. ANCA antigens are not only secreted by preactivated neutrophils but are also presented on their surfaces, and by binding to them and to the FC receptor, ANCAs inappropriately lead to the activation of primed neutrophils and monocytes [82]. Improved adhesion to endothelial cells, dysregulated neutrophil degranulation, oxidative burst, and NETosis contribute to pathogenesis, leading to small vessel obstructions and local tissue damage [83]. ANCA IgG induces multiple signaling pathways in neutrophils that, despite being distinct from normal neutrophil activation, still converge on DGKα. Indeed, DGKα is selectively activated by ANCA and produces an abnormal increase in PA, which is responsible for enhancing neutrophil adhesion, in part, through integrin activation [84], but also by binding to actin-related protein 3 (Arp3) and promoting actin nucleation [58]. If neutrophils are incubated with the DGK inhibitor R59022 before treatment with ANCAs, they exhibit a reduced capacity to release the tissue-damaging content of azurophilic granules such as proteases and myeloperoxidase. This release is restored by PA treatment, demonstrating that DGKα activity also contributes to granule exocytosis. The downstream effectors of PA involved are presently unknown, however, both Ca^2+^ influx and the modulation of membrane fusion are required to restore exocytosis in DGKα-inhibited neutrophils, suggesting a perturbation of intracellular vesicular dynamics. This involvement of DGKα-produced PA in membrane trafficking is expected, as DGKs play a pivotal role in intracellular trafficking. DGKα is known to be required for the assembly of multivesicular bodies, the polarized secretion of exosomes by T cells [85], and also integrin and MHC complex recycling in epithelial cells. DGKα-produced PA mediates the membrane recruitment of several proteins, such as the Rab11 effector RCP and the adaptor MICAL-1 [86,87]. In addition, other DGK isoforms have been implicated in virtually all aspects of intracellular membrane trafficking. This is exemplified by DGKζ interactions with sortin-nexin during endosomal recycling in T cells [88], DGKδ scaffolding activity in the endoplasmic reticulum to the Golgi traffic [89], and DGKθ in synaptic recycling [90]. The finding that stopping PA production by inhibiting DGKα impairs both integrin recycling and secretion suggests that DGKα may represent a viable drug target to reduce tissue injury associated with ANCA-associated vasculitis and other autoimmune disorders where neutrophils play a major role [91].

## 4. DGKs in Respiratory Diseases

While several studies have addressed the biological role of DGKs, their impact on respiratory disease is not completely enlightened and is still under investigation. Asthma and chronic obstructive pulmonary disease (COPD) are characterized by airflow obstruction and represent the most common non-communicable respiratory diseases with an increasing worldwide prevalence and impact on global health.

In relation to asthma, a heterogeneous pattern of inflammatory mechanisms has been identified in the underlying clinical features. The main phenotypes comprise eosinophilic inflammation and Th1 and Th17 neutrophilic inflammation [92].

Eosinophilic inflammation, and consequent type 2 airway inflammation, occurs in a large number of patients, and typically about half of asthmatic patients [93]. The main step is represented by allergic sensitization, where an allergen stimulates dendritic cells that, in the presence of a coactivator, are conducive to the polarization of T helper lymphocytes in T helper 2. Mediators released in type 2 inflammation include interleukin-4, interleukin-5, and interleukin-13 [92], which stimulate and sustain eosinophilic activation. In particular, interleukin-5 is essential for the survival of eosinophils, as they are the only granulocyte that expresses the interleukin-5 receptor [94].

Alongside type 2 inflammation, asthma displays other pathophysiologic patterns underlying its clinical manifestation. Actually, non-eosinophilic asthma has been described both in the young and adults [95]. Green et al. reported the presence of an asthmatic sub-group showing neutrophilic inflammation during the analysis of induced sputum [96]. Here, the trigger allergens and molecular mechanisms are only partially understood. Neutrophilic inflammation is mainly sustained by T helper 1 lymphocyte cytokines, but also T helper 17 cells and type 3 innate lymphoid cells are recognized as playing roles in non-eosinophilic inflammatory processes. Macrophages are intermediate actors of this pathway, mainly releasing neutrophil chemotactic molecules such as CXC motif chemokine ligand 8 (CXCL8) [97]. The protein encoded by this gene is a member of the CXC chemokine family and is a major mediator of the inflammatory response the binding CXC motif chemokine receptor-1 and -2 (CXCR1 and CXCR2).

In relation to eosinophilic inflammation, Wang et al. [98] conducted an interesting study in a murine model of allergic asthma. Plasmids containing the DGKα-encoding gene were inoculated in mice, and asthma was later induced through alum-adsorbed ovalbumin (OVA). Mice “immunized” with the DGKα gene showed significantly lower allergic airway inflammation and reduced eosinophilic infiltration in the lungs when compared to mice that did not receive plasmids with the DGKα-encoding gene. These results suggested a potential critical role of DGKα in allergic and eosinophilic asthma, where elevated levels of DGKα were detected in anergic and non-stimulated T-cells, while reduced levels of DGKα were found in activated T-cells.

DGKα’s role in non-eosinophilic asthma is less clear. The main actors involved in non-eosinophilic asthma are type 1 inflammation cells, including Th1, cytotoxic T-lymphocytes (CTL), and neutrophils. Previous reports have found a reduced transcription of DGKα by IL-2 in CTL [99,100]. CTLs derived from DGKα-deficient mice showed increased IL-2-dependent proliferation and activation as well as an enhanced cytotoxic effect [99]. However, DGKα and ζ double deficiency actually has caused the severe impairment of CD8 T cell-mediated responses, especially in response to bacterial pathogens [101].

Moreover, Ruffo et al. [35] showed that DGKα inhibition restores correct CTL activity in the context of genetic predisposition to EBV-driven lymphohistiocytosis. They found that the persistent activation of DGKα, with subsequent diminishing DAG signaling, lead to the impairment of CTL functions in patients with X-linked lymphoproliferative disease 1 (XLP-1). Patient CTLs lacked restimulation-induced cell death (RICD), a crucial self-regulatory apoptosis program triggered by repeated T cell receptor (TCR) stimulation in order to maintain peripheral immune homeostasis and avoid the pathological accumulation of activated T cells [102]. In murine models of pathology, the inhibition of DGKα was demonstrated to provide a reduction of lymphocytic infiltrates in the liver and bone marrow and of INFγ, suggesting the potential restoration of RICD by CTLs [35].

The complex functions of DGKα have also been reported by Shin et al., who found the inhibitory roles of DGKα and DGKζ in the primary anti-viral immune response, while they appear to promote expansion of viral specific memory cytotoxic T cells during secondary infection [103]. The mechanisms underlying such differential roles of DGK activity in primary and memory anti-viral immunity are unclear, but they may be related to the dual role of DGKs as negative regulators of TCR signaling and also mediators of cytokine responses [104].

Sanjuan et al. investigated the role of DGKα in relation to CD69 expression, one of the early markers of T cell activation in relation to TCR stimulation [105,106]. They demonstrated that DGKα activation is dependent on its translocation from the cytosol to the membrane and leads to reduction in DAG signaling and in CD69 expression. The impact of DGKα in T cell activation needs to be better investigated, but evidence suggests it is not a secondary role in the signaling pathway.

**Figure 3:**
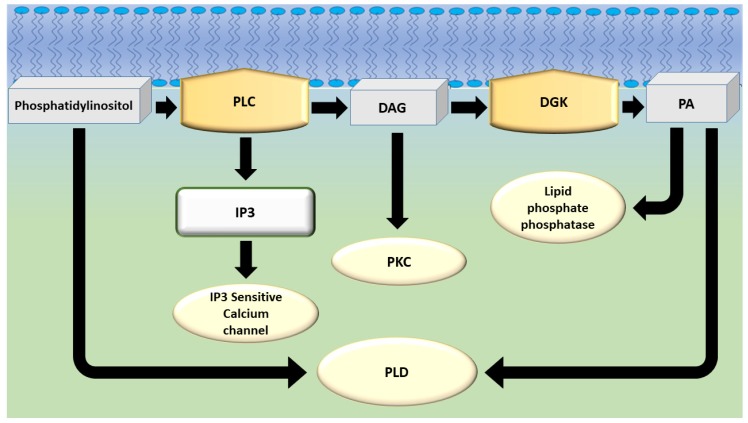
Biochemical pathway showing the interplay between different actors in DAG and phosphatidic acid signaling. DAG: Diacylglycerol; DGK: Diacylglycerol kinase; IP3: Inositol trisphosphate; PA: Phosphatidic acid; PKC: Protein kinase C; PLD: Phospholipase D.

DGK is a physiological modulator of DAG metabolism, limiting PKC activation [107] but also the activity of other C1-containing proteins, such as chiaerins (a family of Rac GAPs) and RASGRPs (a group of Ras GEFs) [108]. However, the phosphorylation of DAG by DGK produces PA, which adds to that produced by the action of phospholipase D (PLD) on glycerophospholipid (Figure 3). PA has long been considered an intermediate step in the resynthesis of phosphatidylinositol, with no specific role in the transduction of receptor-derived signals. On the contrary, PA represents a keystone of intracellular signaling, binding several signaling enzymes such as phosphodiesterases, components of the MAPK cascade, lipid kinases, and p47phox [109]. In addition, PA generation at the membrane induces a negative membrane curvature, which precedes changes leading to either membrane fusion or fission. The regulation of PA generation is, thus, closely linked to the control of exocytosis, endocytosis, and membrane trafficking [110].

One of the most powerful stimuli of chemotaxis for neutrophils is represented by CXCL8, produced mainly by macrophages. Neutrophil migration into the respiratory airway represents a crucial step in COPD progression. Neutrophils secrete serine proteases that act by destroying the alveolar matrix and have a consequent impact on airway architecture. CXCL8 binds the neutrophil chemokine receptors CXCR1 and CXCR2. The signaling cascade includes enhancing intracellular Ca^2+^ levels, with a subsequent release of granule proteins and chemotaxis [111]. Moreover, the stimulation of CXCR1 leads to the increased release of superoxide anions and to the activation of phospholipase D (PLD) and, thus, PA production [112].

Acute exacerbation in COPD patients is one of the key parameters that assesses the severity and the disability of the disease. During acute exacerbation, an increased percentage of neutrophils in the sputum has been observed. The main explanations involve a higher production of chemotactic factors, including leukotriene B4 (LTB4) and CXCL8 [113]. More specifically, Sapey et al. investigated the neutrophil chemotactic response in COPD patients compared to healthy smoking and non-smoking patients and patients with α1-antitrypsin deficiency (1AAD). They found no differences in receptor expression on the neutrophil surface across different populations, but the speed of migration was increased in COPD patient neutrophils with a reduction in migratory accuracy, suggesting impaired intracellular signaling responses [114].

PA has been reported to play a central role in cell adhesion and the migration of leukocytes, especially neutrophils [115,116]. Speranza et al. demonstrated the central role of PA produced by PLD in regulating leukocyte adhesion by the binding of PA to Arp3, which leads to actin polymerization. Adhesion is the obligate first step for rolling leukocytes in blood to anchor to the capillary beds around inflamed or injured tissue, such as that observed in COPD. Moreover, the generation of PA in response to DGK functions participates in receptor endocytosis [117] and regulates transcription [118,119]. Rainero et al. have enlightened the role of DGKα in integrin trafficking modulation. In particular, they reported that the inhibition of DGKα consistently reduced the recruitment of RCP, while the inhibition of PLD had no effect on its recruitment, suggesting that DGKα is more important than PLD as a source of PA to enable RCP functions [86].

**Figure 4:**
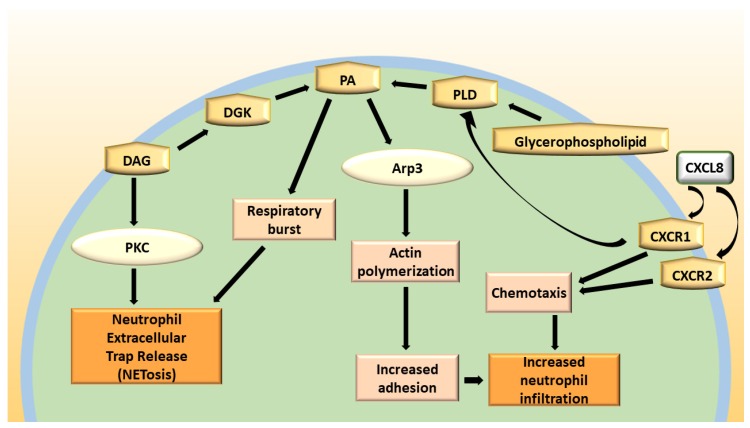
Biochemical pathway in neutrophils showing the signaling cascade and the effects related to DAG and PA activation. DAG: Diacylglycerol; DGK: Diacylglycerol kinase; PA: Phosphatidic acid; PLD: Phospholipase D; PKC: Protein kinase C; Arp3: Actin-related protein 3; CXCL8: C-X-C motif chemokine ligand-8 CXCR1: C-X-C motif chemokine receptor-1; CXCR2: C-X-C motif chemokine receptor-2.

In this direction, the study of Xu et al. investigated the α and β isoforms of PKC in neutrophils, which are the main isoforms involved in maintaining polarization and chemotaxis. In resting neutrophils, they are localized in the cytosol, and, after chemoattractant stimulation, they translocate to the membrane through PLC-dependent signaling, suggesting DAG acts as a major determinant in their regulation [120]. PKC plays a crucial role in the intracellular signaling network to enhance neutrophil actions, in particular, neutrophil extracellular trap (NET) release, a neutrophil-specific form of cell death [56] (Figure 4). The activation of classical PKC by the DAG analog PMA is, indeed, a classical way to induce NET formation through a signaling pathway that comprises calcium fluxes, reactive oxygen species generation by NADPH oxidase, and neutrophil elastase activity [121,122]. Interestingly, neutrophils from chronic granulomatous disease patients with genetic defects in the NADPH oxidase complex do not release NET in response to PMA [121]. Surprisingly, no one has still directly investigated if DGK activity regulates NET release in vitro or in pathological models.

Few data are available in relation to idiopathic pulmonary fibrosis (IPF). IPF is a chronic, progressive, and fatal disease involving lung interstices [123]. The principal pathophysiological features are represented by excessive extracellular matrix depositions that lead to abnormal lung architecture. Impaired damage repair has severe consequences up until respiratory failure [124]. Several elements have been indicated as risk factors to develop IDF. Cigarette smoke and bacterial and viral infections are the main risk factors, followed by radio- and chemo-therapy [125].

Previous studies have suggested the role of PA produced by PLD in the development of kidney fibrosis [126], in particular, its genetic deletion was linked to the attenuated development of kidney fibrosis. Based on this concept, Suryadevara et al. [125] studied PLD KO mice in a murine model of bleomycin-induced lung epithelial injury. Mice without PLD were protected against pulmonary fibrosis, suggesting a significant role of PLD, and, thus, PA in the development of pulmonary fibrosis.

Recently, targeting the diacylglycerol metabolism by DGKζ knockdown or treatment with the DGKα-specific inhibitor R59949 has proven to be beneficial for treating asthma by reducing both inflammation and airway hyperresponsiveness [127]. Ongoing studies are evaluating the impact of DGKα inhibitors in the context of α1-antitrypsin mutations that result in 1AAD. The Glu342→ Lys (Z form) produces inclusion bodies that are retained in hepatocytes rather than secreted in the circulation. Those polymers are cytotoxic to hepatocytes and cause liver damage, while decreased levels in the circulation expose the lung extracellular matrix to degradation by neutrophil elastase [128]. Protein replacement slows the progression of emphysema but does not resolve the disease [129]. As previously reported, DGKα is markedly upregulated throughout neutrophil differentiation and is required for maturation [62]. The activation of DGKα by anti-neutrophil cytoplasmic antibodies inappropriately stimulates neutrophil adhesion, degranulation, and induces an oxidative burst, which contributes to the progression of inflammatory disease [84,91].

## 5. Discussion

The adjustment of DGK activity offers an innovative perspective in the therapeutic manipulation of T cell functions for the treatment of autoimmune pathologies. Having specific information on the mechanisms that sustain DGK isoform-specific regulation in T lymphocytes is crucial for the development of novel tools for pharmacological intervention [100]. While the biological role of DGKs, and DGKα and Dgkζ in particular, is well documented in T cells and in adaptive immunity, few studies have investigated their role in innate immunity. Indeed, neutrophils express multiple DGK isoforms, but only DGKα has been studied in detail, with several in vivo and ex vivo experiments indicating it as a key regulator of neutrophil function. Thus, there is a gap in knowledge on the contribution of the other DGK isoforms to neutrophil biology, as well as on the molecular mechanisms by which the regulation of DAG and PA pools by DGKs influence leukocyte behaviors. This gap mainly is due to an underappreciation of the role of those enzymes outside adaptive immunity, but also due to a lack of suitable tools. Recent advancements in the development of DGKα inhibitors [54,55] and the availability of murine models deficient of specific DGK isoforms will allow the investigation of the suitability of those enzymes as targets for neutrophil-mediated diseases, such as chronic diseases of the respiratory system, and also rare genetic diseases, such as alpha-1-antitrypsin deficiency.

## Abbreviations

Arp3	Actin related protein 3
CXCL8	C-X-C motif chemokine ligand-8
CXCR1	C-X-C motif chemokine receptor-1
CXCR2	C-X-C motif chemokine receptor-2.
COPD	Chronic obstructive pulmonary disease
DAG	Diacylglycerol
DGK	Diacylglycerol kinase
IP3	Inositol trisphosphate
NET	Neutrophil extracellular traps
PA	Phosphatidic acid
PKC	Protein kinase C
PLC	Phospholipase C
PLD	Phospholipase D
RCP	Rab-coupling protein
RICD	Restimulation-induced cell death
TCR	T cell receptor
XLP-1	X-linked lymphoproliferative disease 1
